# Impacts of chronic intermittent ethanol vapor and predator odor on ethanol intake and striatal D_1_ and CB_1_ cannabinoid receptor-expressing medium spiny neurons

**DOI:** 10.3389/fnins.2025.1568952

**Published:** 2025-05-27

**Authors:** Cristiane Aparecida Favoretto, Allyson Nguyen, Gabriela R. Chacon, Amanda J. Roberts, Tali Nadav, Saumya Ranjan, Luisa Becker Bertotto, Fábio Cardoso Cruz, Eric P. Zorrilla

**Affiliations:** ^1^Department of Translational Medicine, The Scripps Research Institute, La Jolla, CA, United States; ^2^Department of Pharmacology, Universidade Federal de São Paulo, São Paulo, Brazil; ^3^Animal Models Core, The Scripps Research Institute, La Jolla, CA, United States

**Keywords:** alcohol, stress, D1-medium spiny neurons, CB1 cannabinoid receptors, *Fos*, corticosterone

## Abstract

**Introduction:**

Stress is a risk factor for ethanol use disorders, which has been modeled by chronic intermittent ethanol (CIE) vapor exposure. Repeated stress alters CB_1_ receptor signaling, which could influence ethanol-related behaviors. Striatal CB_1_ receptors regulate D_1_-medium spiny neurons (D_1_-MSNs), involved in goal-directed behaviors and stress responses. This study tested the hypothesis that predator odor stress interacts with CIE exposure to: (1) increase or accelerate CIE-induced escalation in ethanol intake, (2) increase plasma corticosterone levels, and (3) increase the expression or co-localization of CB_1_ receptors, D_1_-MSNs, and Fos neuronal activation marker in the nucleus accumbens (NAc), dorsomedial (DMS), and dorsolateral (DLS) striatum.

**Methods:**

Male C57BL/6J mice underwent three cycles of 4 days CIE or air exposure, alternated with 5 days ethanol access. During the last two cycles, mice were exposed to predator odor or control bedding before each drinking session. Following the last stressor, brains were processed for RNAscope to label *Cnr1* (encodes CB_1_), *Drd1* (D_1_), and *Fos* (Fos).

**Results:**

As hypothesized, predator odor accelerated the CIE-induced increase in ethanol intake. Contrary to our expectations, CIE did not alter corticosterone levels after the final stressor. Compared to control bedding, predator odor reduced the percentage of *Fos*+ and triple-labeled *Cnr1/Drd1/Fos*+ cells in NAc, but not dorsal striatum. In addition, CIE vs. Air exposure, increased percentages of *Fos*+, double-labeled *Cnr1/Fos*+, *Drd1/Fos*+, and triple *Cnr1/Drd1/Fos*+ cells in the NAc, but not DMS or DLS.

**Discussion:**

Thus, CIE and stress elicited opposite neuroactivational effects on CB_1_-regulated D_1_-MSNs of the NAc. The role of these changes in stress- and CIE-augmented drinking warrants further investigation.

## 1 Introduction

Ethanol is the most widely consumed addictive substance worldwide, and its harmful use is a risk factor for disease, disability, and mortality (World Health Organization [WHO], 2018). Chronic stress is a vulnerability factor for ethanol use disorders (Sinha, 2001, 2008). Perceived threats to safety are considered a fundamental source of stress for humans (Maslow, 1943; Zheng et al., 2016) and are linked to subsequently increased alcohol drinking and stress-related disorders (Grieger et al., 2003; Fullerton et al., 2015; Bradford et al., 2024). Animal models of perceived threats to safety, such as shock, social defeat, maternal separation, and predator stimuli have been widely used to identify the key determinants and underlying mechanisms of negative emotional behaviors and drinking associated with threat-related stress (Volpicelli et al., 1982; van Erp and Miczek, 2001; Logrip and Zorrilla, 2012; Logrip et al., 2012; Steinman et al., 2021; Finn et al., 2024; Ornelas and Besheer, 2024). Here, we employed a naturalistic predator odor exposure model that can increase ethanol intake in mice (Cozzoli et al., 2014; Finn et al., 2018; Finn et al., 2024) to identify striatal neuroadaptations that result from repeated threat.

Chronic intermittent ethanol (CIE) vapor exposure is another predictive model for long-term ethanol-related addictive behaviors (O’Dell et al., 2004; Vendruscolo and Roberts, 2014; Favoretto et al., 2024). Thus, CIE vapor exposure enhances ethanol self-administration in both mice and rats (Valdez et al., 2002; Chu et al., 2007). Additionally, withdrawal from CIE vapor is associated with increased anxiety- and depressive-like behaviors (Zhao et al., 2007; Serrano et al., 2018; Ferragud et al., 2021), as well as heightened aggression in rodents (Kimbrough et al., 2017; Somkuwar et al., 2017), resembling symptoms during ethanol abstinence in humans (Heilig et al., 2010).

Notably, the CIE model can be used to examine how ethanol exposure and stress interact. For example, exposure to repeated forced swim stress increased voluntary ethanol intake in mice subjected to CIE, but not in Air vapor controls (Lopez et al., 2016). Piggott et al. (2020) similarly found that while single-prolonged stress alone did not affect ethanol intake, mice subjected to both stress and CIE exhibited sustained increased ethanol consumption compared to controls. Here, we test the hypothesis that exposure to rat predator odor stress promotes increased ethanol intake in mice exposed to CIE. We also investigated whether a history of CIE affected plasma corticosterone response levels after the final session of predator odor stress.

The endocannabinoid (eCB) system consists of endocannabinoids, such as the fatty acid neuromodulators *N*-arachidonoylethanolamine (AEA) and 2-arachidonoylglycerol (2-AG); their typically G_i/o_ protein-coupled receptors, CB_1_ and CB_2_; and the respective enzymes for their biosynthesis and degradation (Navarrete et al., 2020). CB_1_ receptors, primarily located in axon terminals, mediate retrograde endocannabinoid signaling (Vaughan and Christie, 2005) and are highly expressed in brain regions associated with drug rewards, reinforcement, and addiction, including the striatum (Glass et al., 1997; Wang et al., 2003; Flores et al., 2013).

Chronic ethanol exposure impacts endocannabinoid-CB_1_-mediated pathways (DePoy et al., 2013; Serrano et al., 2018), neuroadaptations that may alter subsequent consummatory, addictive-like, or stress-related behavior (Parylak et al., 2012; Vozella et al., 2023). Stressors activate CB_1_ signaling to mitigate the physiological and behavioral consequences of acute stress exposure (Blasio et al., 2013; Hillard, 2014). Thus, like ethanol, repeated exposure to stress may also alter CB_1_-mediated endocannabinoid signaling, leading to long-term behavioral changes (Romano-López et al., 2012; Amancio-Belmont et al., 2019).

Within the striatum, CB_1_ receptors are found on various neuronal subpopulations, including dopamine D_1_- and D_2_-receptor positive medium spiny neuron (MSN) axon collaterals (Fitzgerald et al., 2012). D_1_-MSNs are implicated in promoting alcohol intake (Cheng et al., 2017), the reinforcing effects of addictive drugs, and responses to stress (Lobo and Nestler, 2011; Francis and Lobo, 2017; Favoretto et al., 2023). Exposure to cycles of voluntary alcohol intake, other addictive substances, and stressors can induce cellular and molecular changes in these subpopulations of striatal neurons that correlate with lasting behavioral outcomes (Lobo and Nestler, 2011; Lobo et al., 2013; Jeanes et al., 2014; Wang et al., 2015; Cheng et al., 2017; Francis and Lobo, 2017; Renteria et al., 2017, 2018). The subregional effects of CIE vapor and stress exposure on CB_1_ expression within striatal D_1_-MSNs are poorly understood. Thus, we here also aimed to test the hypothesis that exposure to CIE vapor and/or predator odor stress influences immediate-early gene and CB_1_ gene expression in D_1_-MSNs within the nucleus accumbens (NAc), dorsomedial striatum (DMS), and dorsolateral striatum (DLS). For that, we used RNAscope to label mRNAs that encode for CB_1_, D_1_, and Fos (*Cnr1*, *Drd1*, and *Fos*, respectively).

## 2 Materials and methods

### 2.1 Animals

Forty adult male C57BL/6J mice (9 weeks old) were purchased from Jackson Laboratories (Bar Harbor, ME) and group-housed (up to five mice per cage) in polycarbonate cages within a temperature-controlled room, maintained on a reverse 12 h light/dark cycle (lights on at 8:00 a.m.). Water and food (Envigo, Teklad Global 2018, 3.1 kcal/g; 24% kcal protein) were provided *ad libitum*, unless otherwise noted. Experimental procedures were carried out after approval by the Institutional Animal Care and Use Committee and met the NIH Guide for the Care and Use of Laboratory Animals.

### 2.2 Experimental design overview—2-bottle choice, CIE, and predator odor stress

Mice were first evaluated for baseline voluntary ethanol intake over 3 weeks in test cages (2 h/day, 5 days/week) using a limited access, two-bottle choice (2BC) paradigm [voluntary “choice” between 15% (w/v) ethanol and water]. For this, mice were individually studied in test cages (not their home cages), equipped with two graduated serological pipets with sipper tubes: one contained 15% ethanol (w/v) diluted in water and the other water. Liquid volumes were visualized before and after each session to calculate ethanol consumption and preference. Drinking sessions began 3–4 h into the dark cycle, and ethanol solutions were prepared weekly. By cage, mice were then assigned to either CIE or air vapor exposure, matched for ethanol intake (g/kg) during the 3^rd^ baseline week.

**FIGURE 1:**
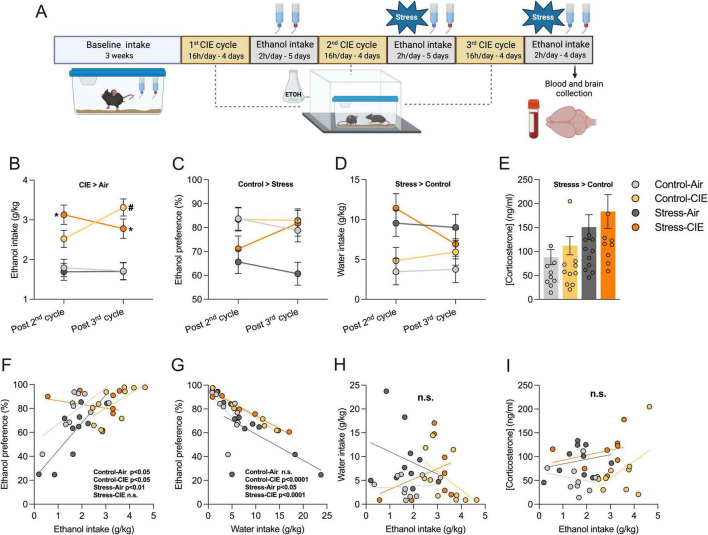
Predator odor stress accelerated chronic intermittent ethanol (CIE)-induced escalation of ethanol intake, reduced ethanol preference, enhanced water intake, and increased plasma corticosterone regardless of CIE. **(A)** Adult male C57BL/6J mice (*n* = 8–10/group) were subjected to 3 cycles of 4 days exposure to CIE vapor or air, alternated with 5 days access to 2-bottle choice voluntary ethanol intake (15% *w/v*). During the last 2 drinking cycles, mice received 30 min of predator odor stress or control cage exposure immediately before each drinking session. Immediately after the last stress/control episode, mice were anesthetized and sacrificed. Brain and blood samples were collected. Brains were sliced and processed for RNAscope *in situ* hybridization. Plasma corticosterone concentrations were determined using an ELISA assay (Created with BioRender.com). **(B)** Estimated marginal means for average ethanol intake (g/kg) following 2^nd^ and 3^rd^ CIE vapor cycles. Mixed Models analysis for CIE (CIE × Air), Stress (Stress × Control), and Time (post 2^nd^ x post 3^rd^ cycle) factors, and average baseline ethanol intake (g/kg) as a covariate detected a main CIE effect (*p* < 0.05; CIE > Air) and CIE × Stress × Time interaction. *Post hoc* test revealed that CIE exposure increased ethanol intake by the 3^rd^ cycle in Stress-CIE (*p < 0.05) and Control-CIE (#*p* < 0.05) groups relative to their respective Air controls. Also, stressed CIE mice presented increased ethanol intake vs. their stressed Air controls earlier - by the 2^nd^ cycle (*p < 0.05). **(C)** Estimated marginal means for average preference for ethanol solution (%). Mixed Models showed main effect of Stress (*p* < 0.05; Control > Stress). **(D)** Estimated marginal means for average water intake (g/kg). Mixed Models detected a significant main Stress effect (*p* < 0.05; Stress > Control). **(E)** Corticosterone concentration (ng/ml) following the 10^th^ stress or control condition episode in mice exposed to CIE or Air. Generalized Linear Models analysis showed a main Stress effect (*p* < 0.05; Stress > Control). **(F–I)** Pearson correlation analyses showing relationships between ethanol intake, ethanol preference, water intake, and corticosterone levels. Significant correlations are indicated. Data are shown as estimated marginal means ± pooled standard errors **(B–D)**. Individual data points are also shown in panel **(E)**.

After receiving a first cycle of 4 days ethanol vapor or air exposure, followed by a 5 days 2BC procedure for voluntary intake, mice were further subdivided into four groups: Control-Air, Stress-Air, Control-CIE, and Stress-CIE (*n* = 8–10 per group; two Stress-CIE mice died, reducing the final sample size of that group to eight) [respective assignments to Stress vs. Control conditions were matched based on ethanol intake (g/kg) during the 1^st^ post-CIE cycle]. They then underwent two additional cycles of ethanol vapor or air exposure, followed by the 2BC procedure. During these 2^nd^ and 3^rd^ cycles, mice in the Stress-Air and Stress-CIE groups were exposed to predator odor stress for 30 min immediately before each 2BC session. Mice in the Control-Air and Control-CIE groups instead were placed in cages with clean bedding for 30 min before the 2BC tests. After the final stress session, mice were rapidly isoflurane-anesthetized and euthanized. Brains were flash-frozen in isopentane under dry ice and stored at −*80*°C. Trunk blood was collected, and plasma was stored at −*80*°C until ELISA for corticosterone levels [*n* = 9–10/group; all mice were included except for one Control-Air animal, which was omitted due to technical limitation (i.e., lack of space on the EIA plate)]. Brain tissue was sectioned using a cryostat, and slices were processed for RNAscope *in situ* hybridization to label mRNAs encoding for CB_1_, D_1_, and Fos (*Cnr1*, *Drd1*, and *Fos*, respectively), as shown in Figure 1A. For RNAscope assay, 4 mice per group (*n* = 4) were randomly selected, and their behavior was representative of the larger cohort (see Supplementary materials, page 2).

### 2.3 Chronic intermittent ethanol (CIE) vapor exposure and 2BC intake

Vapor chambers consisted of acrylic cages holding up to five mice (La Jolla Alcohol Research Inc., La Jolla, CA, United States). Ethanol vapor was generated by introducing 95% ethanol into a vacuum flask at 50°C, with air blowing over it at 11 L/min. Ethanol vapor exposure aimed to achieve blood ethanol concentrations of 100–250 mg/dl. Before exposure, the CIE group received an intraperitoneal (i.p.) injection of ethanol at the dose of 1.75 g/kg and pyrazole at the dose of 68.1 mg/kg (ethanol dehydrogenase inhibitor, Sigma-Aldrich) to reach pharmacologically relevant blood ethanol levels. The Air groups received pyrazole diluted in saline. In each exposure cycle, mice were exposed to vapor for 16 h (3:00 p.m. – 7:00 a.m.; with 8 h off) over 4 consecutive days, followed by 3 days undisturbed in their home cages. In the following days, mice were then given 2 h access to the 2BC procedure (15% ethanol vs. water) for 5 consecutive days. This 4 days CIE or air exposure protocol, followed by the 5 days 2BC procedure, was repeated for 3 cycles (Becker and Lopez, 2004; Lopez and Becker, 2005; Griffin et al., 2009a,2009b; Huitron-Resendiz et al., 2018), with repeated predator stress beginning during the 2^nd^ cycle. Tail blood alcohol levels (BALs) at session end were measured using gas chromatography after each ethanol vapor exposure week. In the Control-CIE group, blood alcohol levels [BALs (mg/dl); *M* ± SEM] were 114.6 ± 6.2, 153.4 ± 8.5, and 222.6 ± 10.3 for the 1^st^, 2^nd^, and 3^rd^ cycles, respectively. In the Stress-CIE group, averages were 90.2 ± 8.6, 150.7 ± 11.7, and 216.2 ± 15.9, respectively.

### 2.4 Exposure to predator odor stress

For the predator odor stress procedure, the experimental mice were removed from their home cages and introduced into polycarbonate cages filled with soiled shavings from adult rats for 30 min, without food and water (Cozzoli et al., 2014). The soiled rat bedding was collected from the cages of adult male and female rats, both same-sex and mate-housed, that had been unchanged for 1 week (most were Wistar strain rats). Control mice were placed in identical cages filled with clean shavings (Envigo/Inotiv 7090 Aspen Sani Chip Bedding) for 30 min, without food and water.

### 2.5 Determination of plasma corticosterone concentrations

Immediately after the final (10^th^) predator bedding or control cage exposure, mice were rapidly anesthetized with isoflurane, decapitated, and trunk blood was collected into wet-ice chilled, EDTA-coated plastic tubes. The blood was centrifuged for 15 min at 3,000 × *g* (4°C) to separate the plasma, which was then stored at −*80*°C until analysis. Corticosterone concentrations were measured in duplicate using the ELISA DetectX^®^ Corticosterone Enzyme Immunoassay Kit (Arbor Assays, Ann Arbor, MI, #K014-H), according to the manufacturer’s instructions. The kit has an intra-assay coefficient of variation (CV) of less than 10%. Plasma samples were diluted 1:400 with the assay buffer provided in the kit.

### 2.6 *In situ* hybridization (RNAscope™)

At sacrifice, brains were also collected, snap-frozen in dry-ice chilled isopentane, and stored at −*80*°C. Brains for a random, behaviorally-representative subset of subjects (*n* = 4/group; see Supplementary materials) were coronally cryosectioned (−*20*°C, 20 μm) and mounted directly on Super Frost Plus slides (Fisher Scientific). Slides were at −*20*°C for ∼10 min and stored at −*80*°C until the *in situ* hybridization assay. For the assay, slides containing brain slices were immersed in 10% neutral buffered formalin (Fisher Scientific) for 20 min at 4°C, washed twice (∼15 s, 1x PBS), and successively dehydrated (5 min/concentration) in 50%, 70%, then 100% ethanol (the latter twice). Slides were dried at room temperature (10 min, ∼22°C), and a hydrophobic pen (ImmEdger Hydrophobic Barrier Pen, Vector Laboratories) was used to make a physical barrier around the brain sections to contain the RNAscope assay solution. We used the Advanced Cell Diagnostics HybEZ Hybridization System. The protease solution (pretreatment solution four) was incubated with sections at room temperature for 20 min. After washing, the sections were incubated with target probes for specific mRNAs (*Fos, Cnr1*, and *Drd1*; catalog #316921, 420721-C2, 461901-C3, respectively) at 40°C, for 2 h, in the HybEZ oven. The sections were then incubated with pre-amplification and amplification probes applying AMP1 (40°C for 30 min), AMP2 (40°C for 30 min), and AMP3 (40°C for 15 min). Then, the sections were incubated with the fluorescently labeled probes far red (TSA vivid fluorophore 650 nm), red (TSA vivid fluorophore 570 nm), and green (TSA vivid fluorophore 520 nm). Finally, sections were incubated for 20 s with DAPI to label nuclei (blue), mounted with ProLong Gold Antifade Mounting medium (Thermo Fisher Scientific, #P36930), and kept at 4°C. Positive and negative control sections were also run.

For DMS and DLS subregions, coronal sections ranging approximately from anteroposterior coordinates +1.1 to 0.38 mm (from bregma, per Franklin and Paxinos, The Mouse Brain in Stereotaxic Coordinates, 3^rd^ edition) were imaged. For the NAc subregion, a narrower interval was considered (+1.1 to 0.62 mm due to the more anterior limit of this structure). Four images (two coronal slices with two brain hemispheres each) were acquired per animal using a laser scanning confocal microscope (Zeiss LSM 710) with the objective of 40 × magnification. Each image comprised four tiles and seven *z*-stacks, each stack with 0.85 μm of thickness. Image acquisition parameters were kept the same across all images. Analysis was performed using QuPath software (Bankhead et al., 2017) - a Random trees (Rtrees) classifier was trained by annotating cells as positive or negative, trained on the selected feature Nucleus: channel# Mean. Classifier performance was then applied to all images in batch mode. Cells were identified based on nuclei labeled with DAPI. The percentage of positive cells for each probe (or a combination of probes) was obtained by dividing the number of cells expressing the probe by the number of nuclei stained with DAPI.

Results are presented as means of the variables of interest calculated for each animal based on the data obtained from each image. An experimenter, blind to treatment groups, excluded images of questionable quality, such as those with high background or channel bleed-through that led to inaccurate cell classification or false-positive signal detection. For the NAc subregion, all available images from four mice were deemed questionable image quality (*n* = 2) or too posterior (*n* = 2) (implicating that the NAc subregion was not present at that coordinate level) and technically excluded from the NAc analysis. Sensitivity analysis showed that exclusion of these mice from the dorsal striatum analysis did not substantively change inferential results (not shown).

**FIGURE 2:**
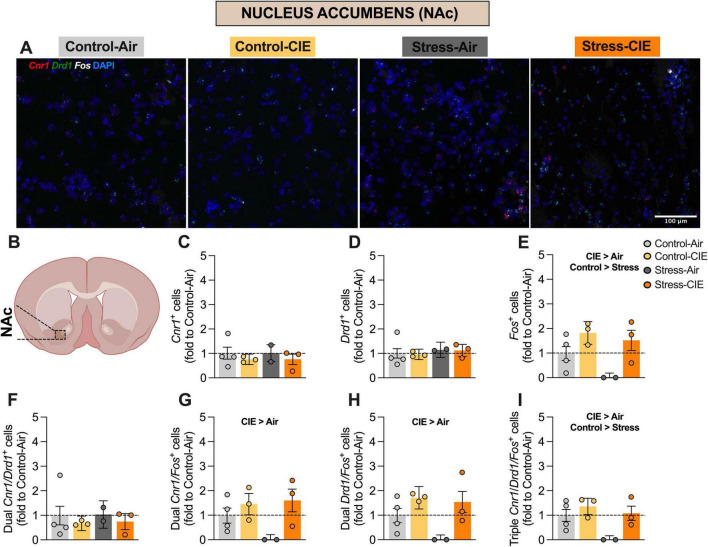
Predator odor stress decreased the percentage of *Fos*+ and *Cnr1/Drd1/Fos*+ cells, while chronic intermittent ethanol (CIE) increased the percentage of *Fos*+, *Cnr1/Fos*+, *Drd1/Fos*+, and triple-labeled cells in the nucleus accumbens (NAc). **(A)** Representative images of NAc cells from the different experimental groups expressing *Cnr1* (in red), *Drd1* (in green), and *Fos* (in white), plus DAPI (in blue). **(B)** Diagram of a mouse brain, with NAc highlighted (Created with BioRender.com). Percentage (fold to Control-Air) of *Cnr1*+cells **(C)**, *Drd1*+ cells **(D)**, *Fos*+ cells **(E)**, dual *Cnr1/Drd1*+ cells **(F)**, dual *Cnr1/Fos*+ cells **(G)**, dual *Drd1/Fos*+ cells **(H)**, and triple *Cnr1/Drd1/Fos*+ cells **(I)**. Generalized Linear Model Analysis found a significant main effect of CIE (CIE > Air) for the percentage of *Fos*+ **(E)**, dual *Cnr1/Fos*+ **(G)**, dual *Drd1/Fos*+ **(H)**, and triple *Cnr1/Drd1/Fos*+ **(I)** cells. Additionally, a main effect of Stress was detected for the percentage of *Fos*+ **(E)** and of triple *Cnr1/Drd1/Fos*+ cells **(I)**. Data are shown as estimated marginal means ± pooled standard errors, with individual data points overlaid as scatter plots.

**FIGURE 3:**
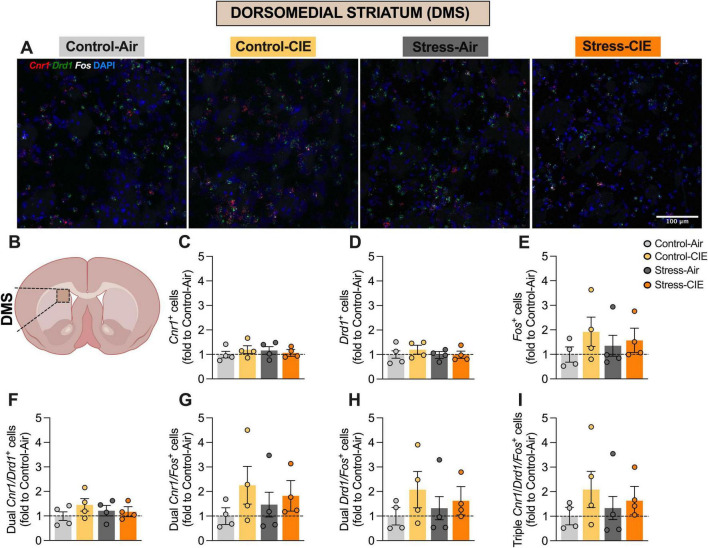
Neither predator odor stress nor chronic intermittent ethanol (CIE) exposure altered the percentage of *Cnr1*+, *Drd1*+, or *Fos*+ cells, or their colocalizations, in the dorsomedial striatum (DMS). **(A)** Representative images of DMS cells from the different experimental groups expressing *Cnr1* (in red), *Drd1* (in green), and *Fos* (in white), plus DAPI (in blue). **(B)** Diagram of a mouse brain, with DMS highlighted (Created with BioRender.com). Percentage (fold to Control-Air) of *Cnr1*+ cells **(C)**, *Drd1*+ cells **(D)**, *Fos*+ cells **(E)**, dual *Cnr1/Drd1*+ cells **(F)**, dual *Cnr1/Fos*+ cells **(G)**, dual *Drd1/Fos*+ cells **(H)**, and triple *Cnr1/Drd1/Fos*+ cells **(I)**. Generalized Linear Model Analysis did not detect any significant effects involving CIE or Stress for any of the assessed variables. Data are shown as estimated marginal means ± pooled standard errors, with individual data points overlaid as scatter plots.

**FIGURE 4:**
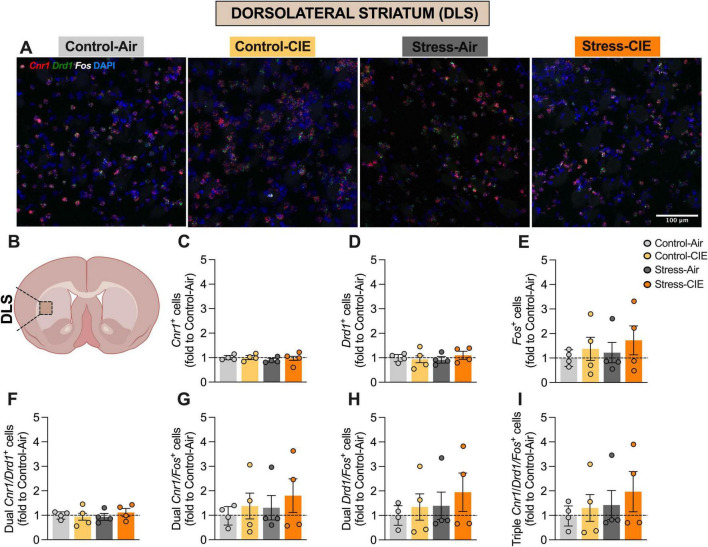
Neither predator odor stress nor chronic intermittent ethanol (CIE) exposure altered the percentage of *Cnr1*+, *Drd1*+, or *Fos*+ cells, or their colocalizations, in the dorsolateral striatum (DLS). **(A)** Representative images of DLS cells from the different experimental groups expressing *Cnr1* (in red), *Drd1* (in green), and *Fos* (in white), plus DAPI (in blue). **(B)** Diagram of a mouse brain, with DLS highlighted (Created with BioRender.com). Percentage (fold to Control-Air) of *Cnr1*+ cells **(C)**, *Drd1*+ cells **(D)**, *Fos*+ cells **(E)**, dual *Cnr1/Drd1*+ cells **(F)**, dual *Cnr1/Fos*+ cells **(G)**, dual *Drd1/Fos*+ cells **(H)**, and triple *Cnr1/Drd1/Fos*+ cells **(I)**. Generalized Linear Model Analysis did not detect any effects of CIE, Stress, or interaction between these factors for any of the assessed variables. Data are shown as estimated marginal means ± pooled standard errors, with individual data points overlaid as scatter plots.

Additionally, images of the cingulate cortex (one tile and seven *z*-stacks, each stack with 0.85 μm of thickness), a region reciprocally connected to the dorsal striatum and NAc, that links emotional and motivational states with action selection and motor control (Reep et al., 2003; Tewari et al., 2016), were analyzed for percentage of *Cnr1*+, *Fos*+, and double-labeled (dual) *Cnr1/Fos*+ cells. Respective results are presented in Supplementary materials (page 1). Representative images shown in Figures 2–4 and Supplementary Figure 1 were uniformly adjusted for brightness for visualization purposes only. Raw images were used for quantitative analyses.

### 2.7 Statistical analysis

Behavioral data were analyzed using Mixed Models to assess the effects of CIE, Stress, and Time, with baseline intake or preference included as a covariate and subject treated as a cluster variable. Significant interactions identified through Mixed Models were further analyzed using Holm *post hoc* tests. Corticosterone and RNAscope data were analyzed using Generalized Linear Models, with CIE and Stress as fixed factors, employing a gamma distribution with a log link function. For RNAscope data, dependent variables were calculated as fold-change (vs. Control-Air) percentages for *Cnr1*+, *Drd1*+, *Fos*+, double-labeled (dual *Cnr1/Drd1*+, *Cnr1/Fos*+, and *Drd1/Fos*+), and triple-labeled (triple *Cnr1/Drd1/Fos*+) cells. A constant value (1) was added to variables containing zero values to allow modeling with the gamma distribution. Following Schmettow (2021), the choice of distribution and link function was based on the data structure — specifically, the use of gamma distribution was appropriate for continuous, non-negative skewed data without upper bounds but with lower boundaries = zero. The gamma distribution yielded a smaller AIC than the Gaussian in 21 out of 25 cases (the 25 comparisons included all RNAscope variables for the NAc, DMS, DLS, and cingulate cortex, as well as corticosterone levels) which is significantly more frequent than the 50% expected by chance (*z* = 3.4, *p* < 0.001; exact binomial *p* < 0.001). Moreover, for each of the striatal subregions, *t*-tests comparing AIC values obtained from Generalized Linear Model analyses that used gamma vs. Gaussian distributions showed that, overall, the mean AIC across analyses for the gamma distribution was significantly lower than that for the Gaussian distribution. This result supports that the cross-sectional data were modeled better by a gamma than Gaussian distribution.

Pearson correlations were performed to evaluate relationships amongst intake, preference, and corticosterone levels. Correlations were conducted in GraphPad Prism (version 10). All other statistical analyses were performed in Jamovi (version 2.3). Statistical significance was defined as *p* ≤ 0.05.

## 3 Results

Predator odor stress accelerated CIE-induced increase of ethanol intake: A three-way (CIE, Stress, Time) mixed models analysis covarying for baseline ethanol intake, revealed a significant main effect of CIE [*B* = 1.21, 95% CI (0.83, 1.60), *p* < 0.001, CIE > Air] and a significant CIE × Stress × Time interaction [*B* = −1.22, 95% CI (−2.03, −0.42), *p* = 0.005]. The model equation was: *Y* = 2.33 + 1.21⋅(CIE)–0.01⋅(Stress) + 0.09⋅(Time) + 0.55⋅(Baseline) + 0.1⋅(CIE × Stress) + 0.25⋅(CIE × Time)–0.52⋅(Stress × Time)–1.22⋅(CIE × Stress × Time). Holm *post hoc* tests to interpret the interaction showed that whereas CIE exposure increased ethanol intake vs. respective Air controls by the 3^rd^ cycle in both the Stress-CIE (*p* = 0.030) and Control-CIE (*p* < 0.001) groups, only Stress-CIE mice exhibited increased ethanol intake compared to Stress-Air by the 2^nd^ cycle (*p* = 0.001). Additionally, Control-CIE mice had higher ethanol intake post-3^rd^ cycle compared to post-2^nd^ cycle (*p* = 0.008), as shown in Figure 1B. Of note, ethanol consumption following the 2^nd^ and 3^rd^ CIE cycles was not compared to drinking after the 1^st^ CIE cycle because exposure to predator odor or control cages only began after the 2^nd^ CIE cycle.

Predator stress reduced ethanol preference by increasing water intake even more than ethanol intake: In terms of ethanol preference (%), a three-way mixed models test covarying for baseline ethanol preference revealed significant main effect of Stress [*B* = −12.43, 95% CI (−20.39, −4.47), *p* = 0.004; Control > Stress], as shown in Figure 1C. The model equation was: *Y* = 75.99 + 7.62⋅ (CIE)–12.43⋅ (Stress) + 0.11⋅ (Time) + 0.32⋅ (Baseline) + 11.34⋅ (CIE × Stress) + 10.09⋅ (CIE × Time) + 5.55⋅ (Stress × Time) + 11.17⋅ (CIE × Stress × Time). Finally, three-way mixed models covarying for baseline water intake also detected a significant main effect of Stress on water intake [*B* = 4.72, 95% CI (1.98, 7.46), *p* = 0.002; Stress > Control], as depicted in Figure 1D. The model equation was: *Y* = 6.86 + 0.83⋅ (CIE) + 4.72⋅ (Stress)–0.92⋅ (Time) + 0.35⋅ (Baseline)–1.90⋅ (CIE × Stress)–1.59⋅ (CIE × Time)−3.19⋅ (Stress × Time)–4.77⋅ (CIE × Stress × Time).

Because ethanol preference and water intake were not normally distributed (*p* < 0.05 Shapiro-Wilk for normality of residuals), we also analyzed data after applying a Box-Cox transformation to ethanol preference and a Log10 transformation to water intake - both resulting in normally distributed residuals (Shapiro-Wilk *p* > 0.05). The inferential outcomes were substantively unchanged, with a main effect of Stress still observed for both measures.

Predator odor stress exposed mice had increased plasma corticosterone levels, regardless of CIE history: A two-way Generalized Linear Model (factors: CIE, Stress) revealed a significant main effect of Stress [*B* = −0.52, 95% CI (−0.87, −0.17), *p* = 0.007]. The model equation was: *Y* = 4.35–0.52⋅Stress + 0.22⋅CIE + 0.04⋅(Stress × CIE). This indicates that stressed mice, regardless of prior CIE or air exposure, exhibited higher mean corticosterone levels after the 10^th^ stress episode compared to control mice (Figure 1E).

Stress-CIE disrupted the direct correlation of ethanol preference to intake: Ethanol intake correlated significantly and directly with ethanol preference (Figure 1F) in the Control-Air (*r* = 0.68; *p* = 0.029), Control-CIE (*r* = 0.66; *p* = 0.037), and Stress-Air (*r* = 0.81; *p* = 0.004), but not Stress-CIE group (*r* = −0.23; *p* = 0.587). Pairwise comparisons of correlations from independent samples showed that the magnitude of correlation within Stress-CIE mice differed significantly from other groups (Table 1).

**TABLE 1:** Chronic intermittent ethanol (CIE) and Stress history alter correlations of ethanol preference to ethanol and water intake.

Correlations comparisons		
Comparison	*z*-value	*P*-value
**Ethanol intake (g/kg) × ethanol preference (%)**		
Control-Air × Stress-Air	−0.55	0.29
Control-Air × Control-CIE	0.08	0.47
Stress-Air × Stress-CIE	2.33	0.01*
Control-CIE × Stress-CIE	1.76	0.04*
**Comparison**	***z*-value**	***P*-value**
**Water intake (g/kg) × ethanol preference (%)**		
Control-Air × Stress-Air	0.27	0.40
Control-Air × Control-CIE	3.66	< 0.002*
Stress-Air × Stress-CIE	2.82	0.002*
Control-CIE × Stress-CIE	−0.28	0.39

Comparative analysis of standardized differences in correlation coefficients (*z*-values) and significance levels (*p*-values) for ethanol intake (g/kg) × ethanol preference (%) and water intake (g/kg) × ethanol preference (%). Group comparisons include Control-Air, Stress-Air, Control-CIE, and Stress-CIE. *p < 0.05.

CIE strengthened the inverse correlation of ethanol preference to water intake: Water intake and ethanol preference (Figure 1G) were strongly inversely correlated in CIE groups (Control-CIE, *r* = −0.99; *p* < 0.0001; Stress-CIE, *r* = −0.99; *p* < 0.0001) and moderately in Air groups (Stress-Air, *r* = −0.66; *p* = 0.039; Control-Air group, *r* = −0.57; *p* = 0.086). Pairwise comparisons of correlations from independent samples showed that the strength of correlation within CIE groups was significantly greater than in their respective Air control group (Table 1).

Ethanol intake not correlated to water or corticosterone levels: Ethanol and water intakes (Figure 1H) did not correlate significantly in any group. There was no significant correlation of ethanol intake (Figure 1I) or preference (not shown) with corticosterone concentration in any group.

Predator odor stress reduced, while CIE exposure increased, the percentage of *Fos*+ and triple *Cnr*1/*Drd*1/*Fos*+ cells in the NAc, but not dorsal striatum: For NAc data, Generalized Linear Model revealed significant main effects of CIE [*B* = 0.64, 95% CI (0.31, 0.97), *p* = 0.005] and Stress [*B* = −0.40, 95% CI (−0.73, −0.07), *p* = 0.044] for the percentage of *Fos*+ cells [model equation: *Y* = 0.67–0.40⋅(Stress) + 0.64⋅(CIE) + 0.58⋅(Stress × CIE)]. For the percentage of dual *Cnr1/Fos*+ cells, Generalized Linear Model analysis found a CIE effect [*B* = 0.59, 95% CI (0.22, 0.94), *p* = 0.013, equation: *Y* = 0.64–0.32⋅(Stress) + 0.59⋅(CIE) + 0.75⋅(Stress × CIE)]. Considering the percentage of dual *Drd1/Fos*+ cells, analysis also detected a main CIE effect [*B* = 0.63, 95% CI (0.28, 0.97), *p* = 0.007, equation: *Y* = 0.66–0.38⋅(Stress) + 0.63⋅(CIE) + 0.63⋅(Stress × CIE)]. Finally, for the percentage of triple *Cnr1/Drd1/Fos*+ cells, Generalized Linear Model analysis revealed main effects of CIE [*B* = 0.45, 95% CI (0.17, 0.74), *p* = 0.014] and Stress [*B* = −0.41, 95% CI (−0.69, −0.12), *p* = 0.022]. The model equation was *Y* = 0.57–0.41⋅(Stress) + 0.45⋅(CIE) + 0.57⋅(Stress × CIE). In all cases, CIE increased expression of the relevant cell population, while exposure to predator odor stress decreased this percentage as compared to control bedding exposure (Figure 2).

No significant effects were detected for the percentage of *Cnr1*+, *Drd1*+ cells, or dual *Cnr1/Drd1*+ cells in the NAc. No significant effects involving CIE or Stress were observed in the DMS or DLS striatal subregions (Figures 3, 4).

No significant effects involving CIE or Stress were seen on the percentage of *Cnr1*+, *Fos*+, and dual *Cnr1/Fos*+ cells in the cingulate cortex (Supplementary Figure 1).

## 4 Discussion

The present study found that predator odor stress accelerated CIE-induced escalation of ethanol intake and reduced the percentage of NAc *Fos*+ and triple-labeled *Cnr1/Drd1/Fos*+ cells. In contrast, CIE vapor exposure increased proportions of *Fos*+, dual *Cnr1/Fos*+ and *Drd1/Fos*+, and triple *Cnr1/Drd1/Fos*+ cells in the NAc. As expected and consistent with previous reports (Griffin et al., 2023; Macedo et al., 2023; Okhuarobo et al., 2020; Xiao et al., 2023), control mice that were not exposed to predator stress developed increased ethanol intake after three cycles of CIE vapor exposure. Supporting our hypothesis, predator odor exposure accelerated the CIE-induced increase in ethanol consumption. Specifically, the Stress-CIE group, but not the Control-CIE group, showed significantly higher ethanol intake compared to the Stress-Air mice after only two cycles of CIE exposure, a difference reflected in a CIE × Stress × Time interaction. This result aligns with findings by Finn et al. (2018), who observed that rat bedding stress exacerbated ethanol intake in male mice with a history of binge drinking, but not in control mice not previously exposed to ethanol. Also similar to the present findings, Rodberg et al. (2017) reported that forced swim stress increased ethanol intake only in mice subjected to CIE vapor, but not in Air controls.

Whereas RNAscope *in situ* hybridization showed that CIE increased the proportion of *Fos*+, double *Cnr1/Fos*+ and *Drd1/Fos*+, and triple *Cnr1/Drd1/Fos*+ NAc cells, Stress specifically decreased *Fos*+ and the triple-labeled population. This result suggests that repeated predator odor may have differentially reduced the activation of the aggregate neuronal population as well as CB_1_-positive D_1_-MSNs, potentially highlighting a unique role for this neuronal subpopulation in stress-related neural adaptations. There is a relative gap in research that addresses the effects of Stress and CIE on CB_1_ receptor-expressing striatal D_1_-MSNs. This is notable given the key role of D_1_-MSNs in modulating rewards-related and stress-associated behaviors through dopaminergic and endocannabinoid signaling (Lobo and Nestler, 2011; Francis and Lobo, 2017; Favoretto et al., 2023). We found that CIE increased and repeated predator odor stress decreased overall *Fos* expression, as well as *Fos* expression in *Cnr1/Drd1*+ cells in the NAc. In contrast, neither manipulation reliably altered *Fos* expression or colocalization with putative D_1_-MSNs or CB_1_-expressing cells in the dorsal striatum, as defined by (co-)expression of *Fos*, *Cnr1*, and/or *Drd1* mRNAs in the DMS or DLS. These results suggest a striatal subregion-specific effect of CIE and predator odor exposure on activation of aggregate neuronal population as well as endocannabinoid-modulated D_1_-MSNs, with a differential impact in the NAc.

It is important to note that this was an exploratory study with a limited sample size. As such, the conclusions should be interpreted with caution and not overstated. We acknowledge that the present study may be underpowered, and therefore we cannot rule out the possibility that effects may become evident in the dorsal striatum or that findings in the NAc could result in CIE × Stress interactions with a larger sample size. Nevertheless, the Bayes Factor scores (BF_10_) for the dorsal striatum were below 1, providing evidence in favor of the null hypothesis in this region (see Supplementary materials). Confirmatory studies with larger cohorts are necessary to validate or refute our findings.

In contrast to positive findings with *Fos*-colocalization in this cellular subpopulation, predator odor and CIE did not reliably change the percentage of cells observed to express *Cnr1* mRNA or co-express *Drd1/Cnr1* in any striatal subregion. Previous stressor paradigms have reportedly yielded both decreases (Hill et al., 2008; Marco et al., 2014; Vangopoulou et al., 2018) and increases (Papilloud et al., 2018; Amancio-Belmont et al., 2019) in *Cnr1* expression or CB_1_ expression/binding within the dorsal and ventral striatum. Few studies have examined predator odor stress effects. Tyler et al. (2020) found with qPCR of NAc punches that a single exposure to TMT (2,4,5-trimethylthiazoline) predator odor reduced *Cnr1* gene expression 4 weeks later in rats. This finding contrasts with our negative results for *Cnr1* cellular localization, perhaps reflecting study differences in the chronicity and nature of the predator odor, species, power, timepoint, *Cnr1* readout, or other experimental conditions.

Prior literature also shows inconsistent effects of ethanol manipulations on striatal CB_1_ or *Cnr1* expression. Increases (Oliva and Manzanares, 2007), decreases (Ortiz et al., 2004; Martín-Llorente et al., 2023), and no changes have been reported (Rubio et al., 2009; Thanos et al., 2011; Favoretto et al., 2025). Here, we found no differences between CIE and Air control mice. Importantly, all mice in our study had a history of 2-bottle choice alcohol drinking, and we cannot rule out that effects of CIE or predator stress on *Cnr1* or *Drd1* expression might have been observed in comparison to alcohol-naïve controls.

Despite increasing ethanol intake, predator odor stress reduced ethanol preference ratios in mice, reflecting an even larger, concurrent, stress-induced increase in water intake. This result resembles findings of Cozzoli et al. (2014), who found that rat bedding stress increased water intake and reduced ethanol preference on the stress exposure day in mice. Thus, the increased drinking was not behaviorally-specific for ethanol, but the mice nonetheless drank more ethanol. Ethanol and water intakes did not correlate with one another, but predator stress and CIE altered the strengths of the correlations of ethanol and water intake to ethanol preference. Specifically, ethanol intake was directly correlated with ethanol preference in all groups except the Stress-CIE group. Conversely, water intake was inversely correlated with ethanol preference more strongly in both CIE groups than in their respective Air controls. The results indicate that predator stress and CIE differently and interactively influence voluntary 2-bottle choice drinking behavior patterns. This may reflect their influences on stress responses, behavioral activation, polydipsia, fluid balance, or other mechanisms (Goto et al., 2014; Gross and Pinhasov, 2016; Zhang et al., 2016).

As expected, corticosterone levels after the 10^th^ stress session were higher than those after the 10^th^ control cage exposure. Cozzoli et al. (2014) previously showed that acute predator odor stress increased corticosterone levels to a comparable degree as restraint stress and tail suspension. Contrary to our initial hypothesis, prior CIE did not potentiate corticosterone levels after the final stress session. Similarly, Finn et al. (2018) found that prior binge drinking did not alter the corticosterone response to predator odor stress. However, a previous study reported that prior CIE exposure enhanced corticosterone responses to a different stressor, forced swim stress, during acute (8 h) but not post-acute (72 h) alcohol withdrawal (Maldonado-Devincci et al., 2016).

Similar to Cozzoli et al. (2014), we found no cross-sectional relationship of corticosterone to ethanol intake or preference on the final day of predator odor stress. Interestingly, Cozzoli et al. (2014) reported that corticosterone levels did instead predict ethanol intake the day after the stressor. Given implicated potential for anticorticosteroid treatments to reduce drinking (Vendruscolo et al., 2012; Somkuwar et al., 2017) further research on temporal dynamics of glucocorticoid responses with ethanol consumption could provide valuable insights.

Several caveats should be considered in interpretation. First, mice were sacrificed 24 h after their last alcohol access, so acute withdrawal may have potentiated or masked effects of predator stress on gene co-localization or corticosterone levels. Indeed, C57BL/6J mice previously showed increased Fos expression in the NAc, but not dorsal striatum during acute alcohol withdrawal (Kozell et al., 2005; Smith et al., 2020). Alcohol withdrawal also reportedly reduced *Cnr1* expression in the dorsal striatum of rats (Marszalek-Grabska et al., 2021) and the NAc of mice (Gasparyan et al., 2023). Future studies that also include alcohol-naïve controls can help determine if access to voluntary 2-bottle choice alcohol drinking modulated effects of predator stress. Our primary rationale for the chosen timepoint of sacrifice was to collect brain and blood samples immediately after the final stress/control cage exposure, which occurred 30 min after the onset of stress. We were aware that both Stress and CIE effects on corticosterone levels and RNAscope outcomes could be influenced by the amount of ethanol consumed, which varied across animals and groups. To minimize this potential confound, we opted to collect samples immediately after the standardized stress/control cage exposure. This ensured that all animals did not acutely have ethanol access at the time of sacrifice, since ongoing ethanol intake (which, as we confirmed, differed across groups) could have introduced bias into our corticosterone and RNAscope data.

Second, we subjected mice to 10 predator odor sessions, so gene expression changes or corticosterone responses may have habituated or sensitized by the final session. Indeed, unlike here, acute exposure to cat odor did increase the number of Fos-expressing cells in the rat caudate-putamen (Staples et al., 2008). On the other hand, Finn et al. (2018) reported no significant changes in corticosterone responses between a 1^st^ and 4^th^ rat bedding stress exposure. Third, other striatal cell types, including *Drd2*/*Adora2a*+ MSNs, other *Drd1*+ MSN subpopulations, interneurons, and glial populations were not studied. Fourth, only RNAs and not proteins were measured. Fifth, the control group used here was an active novel cage change control group, that may itself be stressful; so, other Stress effects might have been seen in comparison to unhandled home cage controls. Sixth, low power due to small size might have yielded false negative findings. Arguing against this, Bayes Factor scores (Keysers et al., 2020) were low for the negative dorsal striatum findings (BFs lower than 0.9, indicating support for the null hypothesis).

In summary, predator odor stress accelerated CIE-induced escalation of ethanol intake but did not potentiate corticosterone response. Repeated predator stress differentially reduced the percentage of NAc, but not dorsal striatum, *Fos*+ and triple-labeled *Cnr1/Drd1/Fos*+ cells. In contrast, CIE vapor exposure more generally increased proportions of *Fos*+, dual *Cnr1/Fos*+ and *Drd1/Fos*+, and triple *Cnr1*/*Drd1*/*Fos*+ cells in the NAc.

Collectively, these findings indicate that stress and alcohol exposure differentially altered activation of accumbal neuronal circuitry, including the CB_1_-regulated D_1_-MSNs population, which could influence rewards-related and appetitive behaviors involved in the development of problematic alcohol use. However, further investigation is needed to confirm these findings and to determine the causal role of these neuroadaptations in driving behavioral changes.

## Data Availability

The raw data supporting the conclusions of this article will be made available by the authors upon request, without undue reservation.
