# Supplementary material for: Impacts of chronic intermittent ethanol vapor and predator odor on ethanol intake and striatal D1 and CB1 cannabinoid receptor-expressing medium spiny neurons
Source: Front Neurosci. 2025 May 27;19:1568952. doi: 10.3389/fnins.2025.1568952 (PMC12149174; doi:10.3389/fnins.2025.1568952)
Supplement: Supplementary file 1 [file Data_Sheet_1.pdf]

Supplementary material

1. RNAscope analysis of percentage of *Cnr1*+, *Fos*+, and double-labeled (dual) *Cnr1/Fos*+ cells in the cingulate cortex (Cg).

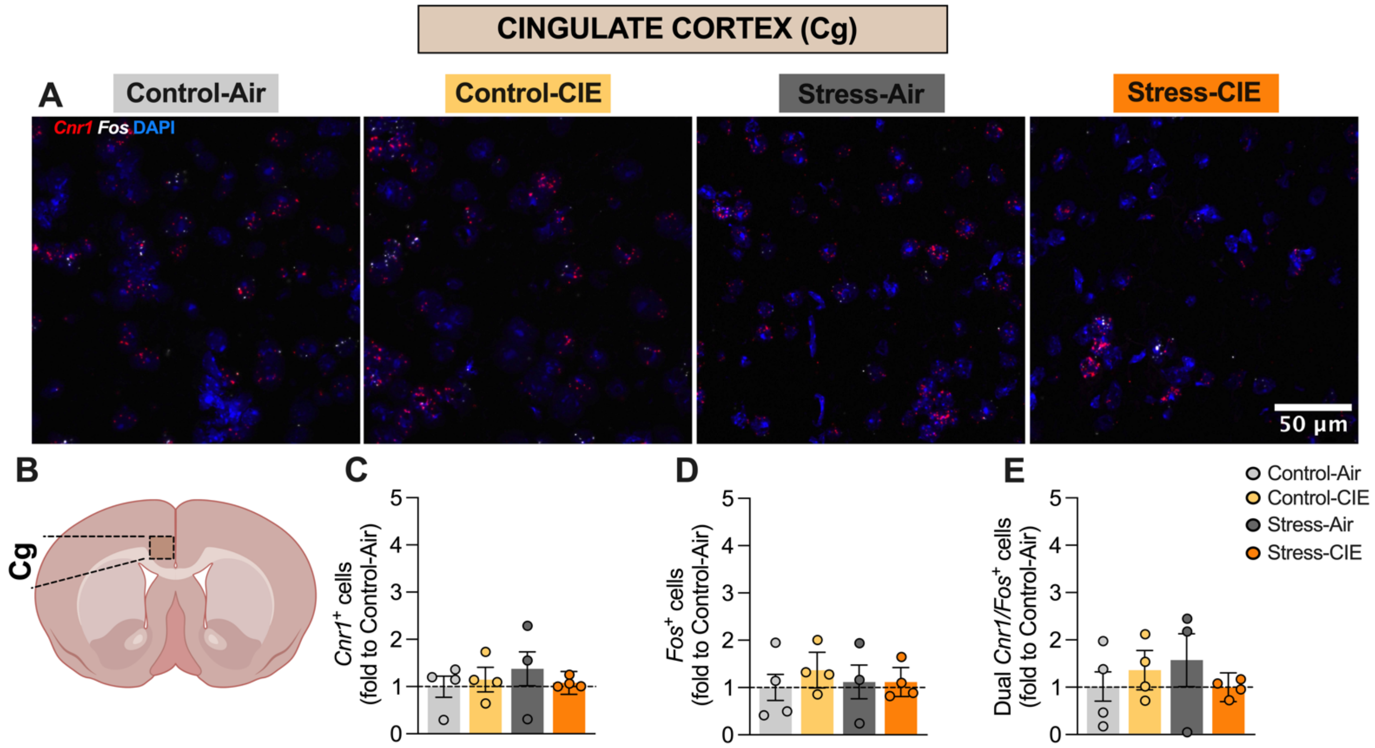

Figure S1: Neither predator odor stress nor chronic intermittent ethanol (CIE) exposure altered the percentage of *Cnr1*+, *Fos*+ or dual *Cnr1/Fos*+ cells in the cingulate cortex (Cg). A) Representative images of Cg cells from the different experimental groups expressing *Cnr1* (in red), and *Fos* (in white), plus DAPI (in blue). B) Diagram of a mouse brain, with Cg highlighted. Created with BioRender.com. Percentage (fold to Control-Air) of *Cnr1*+ cells (C), *Fos*+ cells (D), and dual *Cnr1/Fos*+ cells (E). Generalized Linear Model Analysis did not detect any significant effects involving CIE or Stress for any of the assessed variables. Data are shown as estimated marginal means ± pooled standard errors, with individual data points overlaid as scatter plots.

2. Supplemental comparision of the effect sizes for full dataset vs. RNAscope studied mice to assess representativeness, as determined by Mixed Model analysis of behavioral data.

Ethanol intake – RNAscope studied mice: A three-way mixed model analysis was conducted to examine the effects of CIE, Stress, and Time on ethanol intake covarying for baseline ethanol intake. The model equation was: $Y = 2.29 + 1.31\cdot(\text{CIE}) - 0.20\cdot(\text{Stress}) + 0.10\cdot(\text{Time}) + 0.43\cdot(\text{Baseline}) + 0.06\cdot(\text{CIE} \times \text{Stress}) + 0.28\cdot(\text{CIE} \times \text{Time}) - 0.40\cdot(\text{Stress} \times \text{Time}) - 0.94\cdot(\text{CIE} \times \text{Stress} \times \text{Time})$. Holm post-hoc comparisons between specific groups, in comparison with the full dataset, are shown in Table S1. In addition, estimated *B* coefficients and 95% confidence intervals for main CIE effect and for the three-way interaction (CIE x Stress x Time) are also shown in Table S1.

Table S1: Difference (Diff) and Standard Error (SE) of Holm post-hoc comparisons between specific groups for ethanol intake (g/kg). Estimated *B* coefficients and 95% confidence intervals (CI) for main CIE effect and for the three-way interaction (CIE x Stress x Time).

Comparison	Full dataset	RNAscope mice only
CIE vs. Air (Diff, SE)	1.21, 0.20 (g/kg)	1.31, 0.18 (g/kg)
Stress-Air-2nd cycle vs. Stress-CIE-2nd cycle (Diff, SE)	-1.44, 0.33 (g/kg)	-1.44, 0.35 (g/kg)
Stress-Air-3rd cycle vs. Stress-CIE-3rd cycle (Diff, SE)	-1.08, 0.33 (g/kg)	-1.25, 0.35 (g/kg)
Control-Air-3rd cycle vs. Control-CIE-3rd cycle (Diff, SE)	-1.60, 0.31 (g/kg)	-1.66, 0.35 (g/kg)
Main CIE effect [*B*, (95% CI)]	1.21, (0.83, 1.60)	1.31, (0.95, 1.67)
CIE x Stress x Time effect [*B*, (95% CI)]	-1.22, (-2.03, -0.42)	-0.94, (-2.30, 0.41)

Ethanol preference: A three-way mixed model analysis was conducted to examine the effects of CIE, Stress, and Time on ethanol intake covarying for baseline ethanol preference. The model equation was: $Y = 74.25 + 3.39\cdot(\text{CIE}) - 5.43\cdot(\text{Stress}) + 0.73\cdot(\text{Time}) + 0.68\cdot(\text{Baseline}) + 11.25\cdot(\text{CIE} \times \text{Stress}) + 4.65\cdot(\text{CIE} \times \text{Time}) + 6.40\cdot(\text{Stress} \times \text{Time}) + 8.92\cdot(\text{CIE} \times \text{Stress} \times \text{Time})$.

Holm post-hoc comparisons between specific groups, in comparison with the full dataset, are shown in Table S2. In addition, estimated *B* coefficients and 95% confidence intervals for main Stress effect are also shown in Table S2.

Table S2: Difference (Diff) and Standard Error (SE) of Holm post-hoc comparisons between specific groups for ethanol preference (%). Estimated *B* coefficients and 95% confidence intervals (CI) for main Stress effect.

Comparison	Full dataset	RNAscope mice only
Control vs. Stress (Diff, SE)	12.4, 4.06 (%)	5.43, 5.81 (%)
Main Stress effect [*B*, (95% CI)]	-12.43, (-20.39, -4.47)	-5.43, (-16.82, 5.96)

<u>*Water intake:*</u> A three-way mixed model analysis was conducted to examine the effects of CIE, Stress, and Time on ethanol intake covarying for baseline water intake. The model equation was: $Y = 8.49 + 1.76 \cdot (\text{CIE}) + 2.92 \cdot (\text{Stress}) - 0.93 \cdot (\text{Time}) + 0.97 \cdot (\text{Baseline}) – 1.32 \cdot (\text{CIE} \times \text{Stress}) – 0.79 \cdot (\text{CIE} \times \text{Time}) – 4.51 \cdot (\text{Stress} \times \text{Time}) – 6.03 \cdot (\text{CIE} \times \text{Stress} \times \text{Time})$. Holm post-hoc comparisons between specific groups, in comparison with the full dataset, are shown in Table S3. In addition, estimated *B* coefficients and 95% confidence intervals for main Stress effect are also shown in Table S3.

Table S3: Difference (Diff) and Standard Error (SE) of Holm post-hoc comparisons between specific groups for water intake (g/kg). Estimated *B* coefficients and 95% confidence intervals (CI) for main Stress effect.

Comparison	Full dataset	RNAscope mice only
Control vs. Stress (Diff, SE)	-4.72, 1.40 (g/kg)	-2.92, 1.97 (g/kg)
Main Stress effect [*B*, (95% CI)]	4.72, (1.98, 7.46)	2.92, (-0.95, 6.79)

3. Supplemental Bayesian ANOVA analysis of RNAscope data for the dorsomedial (DMS) and dorsolateral striatum (DLS) subregions.

Table S4. Bayesian two-way ANOVA analyses (factors: CIE and Stress) were conducted in comparison to the null model, for the percentage of *Cnr1*+, *Drd1*+, *Fos*+, *Cnr1/Drd1*+, *Cnr1/Fos*+, *Drd1/Fos*+, and *Cnr1/Drd1/Fos*+ cells in the dorsomedial striatum (DMS). Bayes Factor scores (BF_{10}) are presented for each variable.

Variable	CIE BF_{10}	Stress BF_{10}
Cnr1+	0.45	0.43
Drd1+	0.51	0.52
Fos+	0.73	0.43
Cnr1/Drd1+	0.59	0.43
Cnr1/Fos+	0.85	0.43
Drd1/Fos+	0.77	0.43
Cnr1/Drd1/Fos+	0.87	0.43

Table S5. Bayesian two-way ANOVA analyses (factors: CIE and Stress) were conducted in comparison to the null model, for the percentage of *Cnr1*+, *Drd1*+, *Fos*+, *Cnr1/Drd1*+, *Cnr1/Fos*+, *Drd1/Fos*+, and *Cnr1/Drd1/Fos*+ cells in the dorsolateral striatum (DLS). Bayes Factor scores (BF_{10}) are presented for each variable.

Variable	CIE BF_{10}	Stress BF_{10}
Cnr1+	0.46	0.57
Drd1+	0.47	0.45
Fos+	0.59	0.48
Cnr1/Drd1+	0.46	0.45
Cnr1/Fos+	0.54	0.50
Drd1/Fos+	0.53	0.55
Cnr1/Drd1/Fos+	0.51	0.58
